# Epidemiologic characterization of human papillomavirus (HPV) infection in various regions of Yunnan Province of China

**DOI:** 10.1186/s12879-016-1562-7

**Published:** 2016-05-26

**Authors:** Zulqarnain Baloch, Yuanyue Li, Tao Yuan, Yue Feng, Yanqing Liu, Wenlin Tai, Li Liu, Binghui Wang, A-mei Zhang, Xiaomei Wu, Xueshan Xia

**Affiliations:** Faculty of Life Science and Technology, Kunming University of Science and Technology, Kunming, 650500 China; The Research Center for Molecular Medicine, Kunming, 650500 China; The First Hospital in Yunnan province, Kunming, 650034 China

**Keywords:** Yunnan, Regions, Human papillomavirus, Intercourse, Distribution, Genotype

## Abstract

**Background:**

This study was designed to determine the Human papillomavirus (HPV) prevalence and its distribution of genotypes in various regions of Yunnan Province, China.

**Method:**

In this study, participants were recruited during routine gynecologic examination between Oct 2013 and Feb 2015. A total of 17,898 women were recruited. Polymerase chain reaction was used for detecting the HPV positive samples and HPV geno-array test was used for genotyping.

**Results:**

The overall HPV infection rate (19.9 %) among the south-western women was significantly higher (*P* = 0.001) than that among the north-western (18.0 %), south-eastern (13.3 %), north-eastern (11.1 %) and central women (12.9 %). The high-risk (HR) (18.1 %, *P* = 0.001) and single genotype (16.7 %, *P* = 0.001) infection rates among the South-western women were also significantly higher than those of among the north-western (13.9 %, 11.3 %), south-eastern (11.6 %, 10.5 %), north-eastern (9.6 %, 9.1 %) and central women (10.5 %, 10.0 %), respectively. While, the infections with multiple HPV (4.2 %) genotypes were significantly more common (*P* = 0.001) among women in north-western Yunnan than women in the south-western (1.3 %, 3.1 %), south-eastern (1.7 %, 2.7 %), north-eastern (1.5 %, 2.0 %) and central Yunnan (2.4 %, 2.9 %). A total of 30 HPV genotypes were detected; among them 13 were HR-HPV, 3 were PHR-HPV (Potential High risk), 8 were LR-HPV (Low risk) and six were unclassified. The most common HPV genotypes were HPV-52, 16, 58, 53 in control group, HPV-16, 52, 58, 39 and 53 in CINI (Cervical intraepithelial Neoplasia), HPV-52, 16, 58, 33, 53 and 81 in CINII, HPV16, 58, 18, 52, 81 in CINIII and HPV-16 18, 58, 52 in cervical cancer (CC), respectively. Such variation has also been observed about distribution of HPV genotypes distribution among single and multiple infections.

**Conclusion:**

This study gives an epidemiological estimate of HPV prevalence and different genotype distribution in various region of Yunnan province and further explains its prevalence in different neoplastic lesions. Overall HPV-16, 52, 58, and 18 are the leading HR-HPV genotypes.

## Background

Human papillomavirus (HPV) infections are the most frequently sexually transmitted infections throughout the world. Worldwide, the prevalence of HPV is approximately 11–12 % [1], and 85 % of the infections and 88 % of the deaths caused by this virus occur in developing countries [2]. The prevalence of HPV varies substantially with respect to ethnicity and geographic location [3–5].

Papillomaviruses are non-enveloped, circular, double-stranded DNA viruses with a genome length of 8-kb. More than 200 HPV genotypes have been reported, almost 40 of which are frequently transmitted through intercourse and remain in the genital system. Genital HPV infects most sexually active females at some point during their lives and plays a vital role in the development of cervical cancer [6, 7]. HPV genotypes are classified as high risk or low risk, according to their carcinogenicity. There are about one dozen high-risk HPV genotypes, which are responsible for the formation of malignant lesions [5, 8, 9] while the low-risk genotypes may promote the development of low-grade carcinoma [9, 10]. Epidemiologically, significant geographic variation in HPV genotype distribution has been reported. Generally, the HPV-16 genotype is the most common worldwide, whereas the prevalences of the other genotypes vary from region to region [9]. Several HPV genotypes have been reported to cause cervical cancer, and geographic variation in the frequencies of these genotypes has also been reported [11]. Hence, the assessment of the regional distribution of these genotypes is extremely important for the prevention of cervical cancer.

The prevalence and genotype distributions of HPV are well documented in developed areas of China. The genotypes HPV-16, 18, 52, 58 and 59 are most commonly detected in Chinese women [12–14]. However, the pattern of HPV genotype distribution varies among different regions of the country [15]. Further, due an uneven distribution of cancer registry centres, the actual HPV prevalence is believed to be higher than the reported estimates [4].

Yunnan Province has a unique geographic location, highly complex topography, and large variations in elevation, and it has a diverse climate because of these distinctive features [16]. Yunnan is located at the threshold of the Himalayas. In north-western Yunnan, the mountains are large, the landscape is dry and rugged, and the weather is cold. It shares a border of 4060 km with Myanmar in the west, Laos in the south, and Vietnam in the south-east and the weather is hot in this region. Like its distinct topographical features, the social characteristics of Yunnan Province are also diverse [17]. Although individuals in some ethnic groups live in urban areas with racial admixture, most of them prefer to reside in individual concentrated communities with distinctive socio-cultural practices. Yunnan province is the homeland of a large number of ethnic minorities in China, including the Yi, Bai, Hani, Zhuang, Dai, Miao, Lisu, Hui, Lahu, Va, Naxi, Yao, Tibetan, Jingpo, Blang, Achang, Nu, Pumi, Jino, Benlong, Mongolian and Drung. Each of these ethnicities has its own typical customs, architectural style and unique literature. The Han population is a well-developed ethnic majority in China. It represents the largest population in this region and is equally distributed throughout Yunnan. To our knowledge, there have been no descriptions of the genotype distribution of HPV in Yunnan province. Therefore, the objective of the current study was to explore the epidemiological characteristics of the HPV genotype distributions in various regions of Yunnan Province, China and to further delineate the relationship of HPV genotype distribution with abnormal and normal cytological findings.

## Methods

In this study, participants were recruited during routine gynecologic examination between Oct 2013 and Feb 2015. A Women who met the following criteria were recruited: those who (1) was mentally and physically normal, (2) was aged between above 18 years, (3) was a permanent resident of local region; (3) was not a virgin (4) had not undergone a total hysterectomy; (5) had no history of cervical surgery; (6) had never had pelvic radiation therapy. Initially 18,562 participants were recruited for this study. Sixty-three women excluded with due to invalid test result lesions and 45 were not willing for biopsy while 11 were excluded due to HPV-genotype lesions. A total of 17,898 women from the central region (*n* = 7065) north-west region (*n* = 3772), north-east region (*n* = 3152), south-west region (*n* = 2164) and south-east region (*n* = 1745) were recruited. The study designed and method has been described previously [18] with some modification.

### Experimental ethics

The protocol used in this study was in accordance with the Declaration of Helsinki and was approved by the Ethics Committee at Kunming University of Science and Technology and the Center for Disease Control and Prevention (CDC) in Yunnan Province, China. Written consent was individually obtained from each participant.

### Collection of data and cytological analysis

The women were interviewed at the hospital by trained interviewers using a standardized questionnaire to elicit information on socio-demographic risk factors. After the interview, all women underwent a pelvic examination performed by a senior gynecologist; two cervical scrapings were collected for cytological analysis and detection of HPV DNA. Both conventional smears and liquid-based cytology were performed.

All cytological slides were prepared by two qualified technicians individually. First Cytological slides were read locally by a junior cytopathologist from each geographical site according to the Bethesda classification system. All Pap smears with a diagnosis of atypical squamous cells of undetermined significance (ASC-US) or greater and a 10 % random sample of normal Pap smears were sent to the Research Center for Molecular Medicine, Kunming Yunnan for double cytological review by an expert senior cytopathologist who was blinded to the original diagnosis. Additionally, the slides read by the junior cytopathologist were used only for screening. The final diagnosis was the diagnosis of senior cytopathologist.

### Histo-pathological analysis

During colposcopy, the cervix was divided into four parts and each part was observed individually. All visually abnormal areas were biopsied. Every part with normal colposcopic impressions had one random biopsy collected at the squamo-columnar junction. Endocervical curettage was then performed. All cervical biopsies were performed with standard 2 mm POI biopsy forceps, which allows rapid healing of the biopsy sites and minimizes patient distress. Histological slides were reviewed by two senior pathologists from Yunnan First Peoples Hospital.

The cervical intraepithelial neoplasia grading and cervical cancer was diagnosed according to the World Health Organization classification system *(CIN I–III)* [19].

### HPV genotyping

Samples that tested positive for β-globin were analysed by PCR amplification of HPV DNA. HPV-positive samples were confirmed by PCR with universal L1 primer MY09/11 and GP5/6 systems [20]. DNA from HeLa and Caski cell lines was used as positive controls, and mixtures without sample DNA were used as negative controls. HPV genotypes were determined using an HPV GenoArray Test Kit (Hybribio, Chaozhou, China), according to the manufacturer’s instructions. Geno-Array is an L1 consensus primer-based PCR assay [21] that is capable of amplifying 23 HPV genotypes, including 13 HR-HPV genotypes (HPV-16, 18, 31, 33, 35, 39, 45, 51, 52, 56, 58, 59, and 68), 3 PHR-HPV genotypes (HPV-53, 66, and 81) and 7 low-risk HPV (LR-HPV) genotypes (HPV-6, 11, 42, 43, 44 and 61). The assay was conducted according to the manufacturer’s recommendations. PCR was performed in a reaction volume of 25 μl containing 5 μl of DNA template, 19.25 μl of the provided master mix, and 0.75 μl DNA Taq polymerase, using a Perkin-Elmer GeneAmp PCR System 9700 (Applied Biosystems) The amplification procedure was performed as follows: 9 min of denaturation at 95 °C, followed by 40 cycles of 20 s of denaturation at 95 °C, 30 s of annealing at 55 °C, 30 s of elongation at 72 °C, and a final extension for 5 min at 72 °C.

All the samples that were identified as positive through PCR were genotype with two methods. First we run Geno-Array test and secondly we performed direct sequencing. For the DNA sequencing, new PCR was run with reaction mixture 50 μL containing 6 μL of the DNA extract, 1 μL of 10 μmolar MY09 primer, 1 μL of 10 μmolar MY11 primer and 25 μL of the provided master mixture, and 15 μL dH2O. The consensus primer products were separated by electrophoresis on a 2 % agarose gel and purified with Tiangel PCR purification kit. The DNA was then directly sequenced using the ABI PRISM Big Dye Terminator Cycle Sequencing Ready Reaction (Invitrogen, Beijing, CN) on an ABI 310 DNA analyzer. The nucleotide sequences were aligned and compared with those of known HPV genotypes available through Genbank by using the BLAST 2.0 software server (http://blast.ncbi.nlm.nih.gov/Blast.cgi). The sample was identified as a particular HPV genotype if the sequence was 95 % homologous with the reference standard.

### Statistical analysis

The Chi-square test was performed to compare the differences in HPV genotype distribution among the histo-pathologically normal and abnormal samples and among the various regions. Data analysis was performed using SPSS 20.0 (SPSS, Chicago, USA). All statistical tests were two-sided; *P* values < 0.05 were considered statistically significant.

## Results

### Population characteristics

**Fig. 1:**
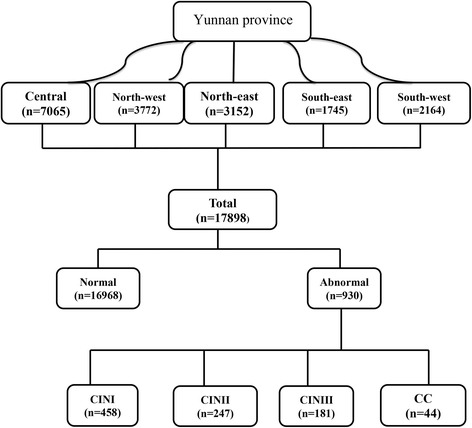
Overview of study population

All of the participants were recruited from various regions of Yunnan province, China (Table 1 and Fig. 1 show the regions inhabited by the study participants). The median age of the 17,898 women included in this study was 38 years, with a range of 18 to 93 years (mean = 40.5 years, SD = 8.79 years, 95 % CI, 40.37–40.63). A total of 75.2 % women were Han, and 24.8 % were of other ethnicities. In addition, 38.3 % of the women came from rural areas, and 61.7 % were from urban areas. Most of the women (82.3 %) were married and reported having a single sexual partner, and the mean number of years of sexual activity was 17. Only 57.7 % of the women had delivered a single baby. A summary of the participants’ characteristics is shown in Table 1. Of the 17,898 women, 16,968 (94.8 %) had normal, 458 had CIN1, 247 had CIN2, 181 had CIN3, and 44 had CC (Table 3).

**Table 1:** The characteristics of HPV infected individuals

	Overall	Central	North-west	North-east	South-west	South-east
	(*n* = 17898)	(*n* = 7065)	(*n* = 3772)	(*n* = 3152)	(*n* = 2164)	(*n* = 1745)
Normal	16968 (94.8)	6704 (94.9)	3570 (94.6)	2984 (94.7)	2062 (95.3)	1648 (94.4)
CINI	458 (2.5)	173 (2.4)	97 (2.8)	85 (2.7)	51 (2.3)	52 (3.0)
CINII	247 (1.4)	103 (1.4)	56 (1.5)	40 (1.3)	23 (1.1)	25 (1.4)
CINIII	181 (1.0)	64 (0.9)	37 (1.0)	36 (1.1)	25 (1.5)	19 (1.1)
CC	44 (0.2)	21 (0.3)	12 (0.3)	7 (0.2)	3 (0.1)	1 (0.05)
Ethnic						
Han	13465 (75.2)	5499 (77.8)	2841 (75.3)	2157 (68.4)	1658 (76.6)	1310 (75.1)
Others	4433 (24.8)	1566 (22.2)	931 (24.7)	995 (31.6)	506 (23.4)	435 (24.9)
Area						
Rural	6849 (38.3)	2695 (38.1)	1462 (38.8)	1176 (37.3)	820 (37.9)	696 (39.9)
Urban	11049 (61.7)	4370 (61.9)	2310 (61.2)	1976 (62.7)	1344 (62.1)	1049 (60.1)
Education						
Graduate	4399 (24.6)	1780 (25.2)	897 (23.8)	847 (26.9)	422 (19.5)	453 (25.9)
High	3841 (21.5)	1533 (21.7)	845 (22.4)	684 (21.7)	408 (18.8)	371 (21.3)
Middle	5564 (31.1)	2282 (32.3)	1114 (29.5)	892 (28.3)	733 (33.9)	543 (31.1)
Primary	2370 (13.2)	807 (11.4)	515 (13.6)	368 (11.3)	481 (22.2)	199 (11.4)
Illiterate	1724 (9.6)	663 (9.4)	401 (10.6)	361 (11.4)	120 (5.5)	179 (10.2)
Profession						
Farmer	3886 (21.7)	1321 (18.7)	730 (19.3)	643 (20.4)	663 (30.7)	529 (30.3)
Government	3489 (19.5)	1407 (19.9)	663 (17.6)	602 (19.1)	496 (22.9)	321 (18.4)
Others	2410 (13.5)	968 (13.7)	547 (14.5)	351 (11.1)	307 (14.2)	237 (13.4)
Private	3918 (21.9)	1613 (22.8)	882 (23.4)	704 (22.3)	381 (17.6)	338 (19.4)
Services	2689 (15.9)	1141 (16.1)	619 (16.4)	497 (15.8)	253 (11.7)	179 (10.2)
No work	1506 (8.4)	615 (7.8)	331 (8.8)	355 (11.3)	64 (2.9)	141 (8.1)
Smoke						
Yes	7619 (42.6)	2981 (42.2)	1565 (41.5)	1409 (44.7)	903 (41.7)	761 (43.6)
No	10279 (57.4)	4084 (57.8)	2207 (58.5)	1743 (55.3)	1261 (58.3)	984 (56.4)
Drinking						
Yes	11203 (62.6)	4759 (67.4)	1613 (42.8)	2183 (69.2)	1469 (67.9)	1179 (67.6)
No	6695 (37.4)	2306 (32.6)	2159 (57.2)	969 (30.7)	695 (32.1)	566 (32.4)
Material status						
Married	14675 (82.0)	5921 (83.3)	3078 (81.6)	2478 (78.6)	1799 (83.1)	1399 (80.2)
Single	3223 (18.0)	1144 (16.2)	694 (18.4)	674 (21.3)	365 (16.9)	346 (19.8)
Pregnancy						
Single	10334 (57.7)	4511 (63.8)	2033 (53.9)	1653 (52.4)	1187 (54.8)	950 (54.4)
Multiple	7564 (42.3)	2554 (36.2)	1739 (46.1)	1499 (47.5)	977 (45.1)	795 (45.4)
Age groups						
<35 years	6582 (36.8)	2779 (39.3)	1452 (38.5)	1092 (34.6)	759 (35.1)	500 (28.6)
36–45	6941 (38.9)	3195 (45.3)	957 (25.4)	1115 (35.4)	710 (32.8)	964 (55.2)
>46 years	4375 (24.4)	1091 (15.4)	1363 (36.1)	945 (30.0)	695 (32.1)	281 (16.1)

*n* number

### HPV genotype distribution by region

The overall HPV infection rate (19.9 %) among the south-western women was significantly higher than that among the north-western (18.0 %), south-eastern (13.3 %), north-eastern (11.1 %) and central women (12.9 %). The high-risk (HR) (18.1 %) and single genotype (16.7 %) infection rates among the South-western women were also significantly higher than those of among the north-western (13.9 %, 11.3 %), south-eastern (11.6 %, 10.5 %), north-eastern (9.6 %, 9.1 %) and central women (10.5 %, 10.0 %), respectively. While, the infections with other HPV (4.1 %) and with multiple HPV (4.2 %) genotypes were significantly more common (*P* = 0.001) among women in north-western than women in the south-western (1.3 %, 3.1 %), south-eastern (1.7 %, 2.7 %), north-eastern (1.5 %, 2.0 %) and central women (2.4 %, 2.9 %). The HPV genotype distribution data by region is shown in Table 4. The prevalence of HPV-16 (5.9 %) and HPV-58 (3.5 %) were significantly (*P* = 0.001, *P* = 0.001) more frequent in the southwest region compared with those in the central, north-east, south-east, and north-west regions. With the exception of the above-mentioned genotypes, no significance differences in the distributions of any of the other HPV genotypes were observed among the five regions of Yunnan province (Table 2).

**Table 2:** HPV Prevalence and genotype distribution among various regions of Yunnan province

Variable	Overall	Central	North-east	North-west	South-east	South-west	*P*-Value
	(*n* = 17898)	*n* = 7065	*n* = 3152	*n* = 3772	*n* = 1745	*n* = 2164	
Overall^a^	14.0 (13.5–14.5	12.9 (12.1–13.7)	11.1 (10.0–12.2)	18.0 (16.8–19.2)	13.3 (11.7–14.9)	19.9 (18.2–21.6)	0.001
High risk^a^	12.2 (–11.7–12.7)	10.5 (9.8–11.2)	9.6 (8.6–10.6)	13.9 (12.0–14.2)	11.6 (10.1–13.1)	18.1 (16.5–19.7)	0.001
Others^a^	1.8 (1.6–2.0)	2.4 (2.04–2.7)	1.5 (1.1–1.9)	4.1 (3.5–4.7)	1.7 (1.1–2.3)	1.3 (0.8–1.8)	0.001
Single infection^a^	11.0 (10.5–11.4)	10.0 (9.3–10.7)	9.1 (8.1–10.1)	11.3 (10.3–12.3)	10.5 (9.1–11.9)	16.7 ((15.1–18.3)	0.001
Multiple infection^a^	3.0 (2.8–3.2)	2.9 (2.5–3.3)	2.0 (1.5–2.5)	4.2 (3.6–4.8)	2.7 (1.9–3.5)	3.1 (2.4–3.8)	0.001
Unidentified^a^	1.1 (0.9–1.2)	0.7 (0.5–0.9)	0.8 (0.5–1.1)	2.2 (1.7–2.7)	0.9 (0.5–1.3)	0.9 (0.5–1.3)	0.009
HR-HPV							
HPV-52^a^	3.1 (2.9–3.3)	3.4 (3.0–3.8)	2.4 (1.9–2.9)	3.3 (2.7–3.9)	3.0 (2.2–3.8)	3.1 (2.4–3.8)	0.15
HPV-16^a^	3.4 (3.1–3.7)	3.0 (2.8–3.2)	2.4 (1.9–2.9)	3.5 (2.9–4.1)	3.4 (2.5–4.2)	5.9 (4.9–6.9)	0.001
HPV-58^a^	2.1 (1.9–2.3)	1.9 (1.6–2.2)	1.4 (1.0–1.8)	2.3 (1.8–2.8)	1.5 (0.9–2.1)	3.5 (2.7–4.3)	0.001
HPV-18^a^	1.2 (1.0–1.3)	1.1 (0.9–1.3)	1.4 (1.0–1.8)	1.4 (1.0–1.8)	13 (0.7)	1.5 (1.0–2.0)	0.16
HPV-51	120 (0.7)	49 (0.7)	18 (0.6)	23 (0.6)	-	30 (1.4)	0.2
HPV-39	118 (0.6)	45 (0.6)	13 (0.4)	34 (0.9)	17 (1.0)	9 (0.4)	0.69
HPV-33	124 (0.7)	44 (0.6)	20 (0.6)	52 (1.4)	-	8 (0.4)	0.35
HPV-68	122 (0.7)	43 (0.6)	19 (0.6)	31 (0.8)	9 (0.5)	20 (0.9)	
HPV-56	60 (0.3)	32 (0.4)	4 (0.1)	8 (0.2)	8 (0.4)	8 (0.4)	
HPV-59	56 (0.3)	27 (0.4)	3 (0.1)	19 (0.5)	5 (0.3)	2 (0.09)	
HPV-31	63 (0.3)	23 (0.3)	9 (0.3)	11 (0.3)	7 (0.4)	13 (0.6)	
HPV-35	(12) 0.07	7 (0.01)	5 (0.1)	-	-	-	
HPV-45	12 (0.07)	3 (0.04)	5 (0.1)	-	4 (0.2)	-	
PHR-HPV							
HPV-53	176 (1.0)	75 (1.1)	33 (1.0)	32 (0.8)	25 (1.4)	11 (0.5)	
HPV-81	108 (0.6)	42 (0.6)	12 (0.4)	23 (0.6)	13 (0.7)	18 (0.8)	
HPV-66		15 (0.2)	9 (0.3)	16 (0.4)	10 (0.6)	18 (0.8)	
LR-HPV							
HPV-11	68 (0.4)	14 (0.2)	-	7 (0.2)	6 (0.3)	5 (0.2)	
HPV-6	16 (0.09)	9 (0.1)	-	4 (0.1)	3 (0.2)	-	
HPV-61	30 (0.2)	-	9 (0.3)	5 (0.1)	7 (0.4)	9 (0.4)	
HPV-43	15 (0.08)	7 (0.1)	-	-	-	8 (0.4)	
HPV-55	7 (0.04)	6 (0.08)	1 (0.03)	-	-	-	
HPV-44	12 (0.07)	5 (0.07)	-	-	-	7 (0.3)	
HPV-42	9 (0.05)	4 (0.06)	-	-	3 (0.2)	2 (0.09)	
HPV-40	4 (0.02)	-	4 (0.1)	-	-	-	
HPV-67	1 (0.005)	-	1 (0.03)	-	-	-	
HPV-69	1 (0.005)	-	1 (0.03)	-	-	-	
HPV-70	2 (0.01)	2 (0.03)	-	-	-	-	
HPV-71	3 (0.002)	3 (0.04)	-	-	-	-	
HPV-82	6 (0.03)	1 (0.02)	-	-	2 (0.1)	3 (0.1)	
HPV-83	2 (0.01)	-	-	1 (0.02)	-	1 (0.05)	

*n* number, ^a^95% Confidence interval

### HPV genotypes and histo-pathological grades

**Fig. 2:**
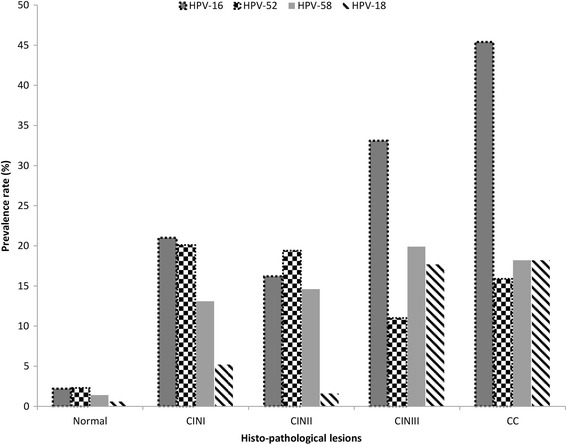
Distribution of leading HPV genotypes among various histo-pathological lesions

The prevalence of overall, HR-HPV, and single genotypes infection significantly increased as the infection progressed in advanced lesions. While, the prevalence of multiple genotypes infection and other genotypes were decreased as the infection progressed in advanced lesions but the difference was not significant. HPV-16, 58, 52, and 18 were significantly more frequent in advance abnormal cervical lesion that those of other genotypes (Fig. 2). The distribution of HPV genotypes according to cervical histo-pathological stage is shown in Table 2. A total of 23 HPV genotypes were detected in the histo-pathologically abnormal samples with different prevalence rates. The six most common HPV genotypes detected in the CINI samples were HPV-16, 52, 58, 53, 39, and 81. In the CINII samples, HPV-52 was the most prevalent, followed by HPV-16, 58, 33, 81, and 51. However, in the CINIII and CC samples, HPV-16 was more prevalent, followed by HPV-58, 18, and 52. A total of 30 HPV genotypes were detected in the normal group, the most common of which were HPV-52, followed by HPV-16, 58, 53, 18, 51, 68 and 81 (Table 3).

**Table 3:** Prevalence of Human Papillomavirus (HPV) and its genotype distribution among normal and histo-pathological abnormal lesion

Variable	Normal	CIN1	CIN2	CIN3	SCC	*P*-Value
	(*n* = 16968)	(*n* = 458)	(*n* = 247)	(*n* = 181)	(*n* = 44)	
overall^a^	10.0 (9.5–10.4)	81.2 (77.6–84.8)	86.2 (81.9–90.5)	94.5 (91.2–97.8)	95.4	0.001
High risk^a^	9.1 (8.7–9.5)	64.0 (59.6–68.4)	65.2 (59.3–71.1)	80.1 (74.3–85.9)	88.6 (79.2–98.0)	0.001
Others^a^	166 (1.0)	17.2 (13.8–20.6)	21.0 (15.9–26.1)	14.4 (9.3–19.5)	6.8	0.17
Single^a^ infection^a^	8.3 (7.9–8.7)	56.8 (51.9–60.9)	55.5 (49.3–61.7)	72.4 (65.9–78.9)	77.3 (64.9–89.7)	0.001
Multiple infection^a^	1.8 (1.6–2.0)	24.4 (20.5–28.3)	30.8 (25.1–36.5)	22.1 (16.1–28.1)	18.2 (6.8–29.6)	0.5
Unidentified^a^	0.7	7.0 (4.7–9.3)	8.1 (4.7–11.5)	6.6 (3.0–10.2)	9.1 (0.6–17.6)	
HR-HPV						
HPV-16^a^	2.2 (1.9–2.4)	21.0 (17.3–24.7)	16.2 (11.6–20.8)	33.1 (26.3–39.9)	45.4 (30.7–60.1)	0.001
HPV-52^a^	2.3 (2.1–2.5)	20.1 (16.4–23.8)	19.4 (14.5–24.3)	11.0 (6.5–15.5)	15.9 (5.1–26.7)	0.025
HPV-58^a^	1.4	13.1 (10.0–16.2)	14.6 (10.2–20.0)	19.9 (14.1–25.7)	18.2 (6.8–29.6)	0.04
HPV-18^a^	0.6	5.2 (3.2–7.2)	1.6 (0.03–3.2)	17.7 (12.1–23.3)	18.2 (6.8–29.6)	0.001
HPV-51	92 (0.5)	16 (3.5)	12 (4.8)	-	-	
HPV-68	84 (0.5)	23 (5.0)	8 (3.2)	7 (3.9)	-	
HPV-33	80 (0.5)	20 (4.4)	20 (8.1)	4 (2.2)	-	
HPV-39	76 (0.4)	28 (6.1)	8 (3.2)	4 (2.2)	-	
HPV-31	48 (0.3)	6 (1.3)	4 (1.6)	5 (2.8)	-	
HPV-56	40 (0.2)	8 (1.7)	8 (3.2)	4 (2.2)	-	
HPV-59	40 (0.2)	8 (1.7)	-	4 (2.2)	4 (9.1)	
HPV-45	4 (0.02)	6 (1.3)	-	2 (1.1)	-	
HPV-35	9 (0.05)	3 (0.6)	-	-	-	
PHR-HPV						
HPV-53	128 (0.7)	28 (6.1)	20 (8.1)	-	-	
HPV-81	52 (0.3)	24 (5.2)	19 (7.7)	12 (6.6)	-	
HPV-66	44 (0.2)	8 (1.7)	8 (3.2)	4 (2.2)	3 (6.8)	
Others						
HPV-61	24 (0.1)	-	3 (1.2)	-	-	
HPV-11	24 (0.1)	-	4 (1.6)	4 (2.2)	-	
HPV-6	12 (0.07)	-	-	4 (2.2)	-	
HPV-43	8 (0.05)	4 (0.9)	-	3 (1.6)	-	
HPV-44	8 (0.05)	4 (0.9)	-	-	-	
HPV-42	4 (0.02)	2 (0.4)	-	3 (1.6)	-	
HPV-55	4 (0.02)	3 (0.6)	-	-	-	
HPV-40	4 (0.02)	-	-	-	-	
HPV-67	1 (0.005)	-	-	-	-	
HPV-69	1 (0.005)	-	-	-	-	
HPV-70	2 (0.01)	-	-	-	-	
HPV-71	3 (0.01)	-	-	-	-	
HPV-82	6 (0.03)	-	-	-	-	
HPV-83	2 (0.01)	-	-	-	-	

*n* number, ^a^95 % Confidence interval

In this study, three PHR-HPV genotypes (HPV-53, 66, and 81) were also detected, and they were highly prevalent in the CIN1 and CIN2 samples. Only three CC samples were found to have a co-infection with the HPV-66 and HR-HPV genotypes. Interestingly, eight single and multiple LR-HPV infections were detected in both the normal and abnormal cytology groups. Some of the unclassified HPV genotypes, such as HPV-67, 69, 70, 71, 82 and 83, were also found in the control group. Table 3 shows the genotype distributions among the abnormal cytology and control groups.

### HPV genotypes and infection

The distribution of HPV genotypes according to the type of infection is shown in Table 3. A total of 16,968 women had normal cytological (normal group) results, and of them, 8.3 % (1402/16968) were infected with a single HPV genotype. In contrast we identified the presence of HPV DNA in 60.4 % (562/930) of the women in the abnormal histo-pathological group. Multiple genotype infections were the most common, and the rate of this type of infection was significantly higher (Table 2, *P* = 0.001) in the abnormal histo-pathological group (236/930, 24.4 %) compared with that in the normal group (1.8 %, 304/16968). The proportion of uncharacterised infection was also higher in the abnormal histo-pathological group (7.1 %, 68/930) compared with that in the normal group (0.75 %, 128/16968). The distribution of HPV genotypes according to the type of infection is shown in Table 3. In cases of single infection, HPV-16 (abnormal histo-pathological group = 136/562, 24.2 %; normal group = 280/16968, 1.6 %; *P* = 0.001), 18 (abnormal histo-pathological group = 48/562, 8.5 %; normal group = 84/16968, 0.5 %; *P* = 0.003), 33 (abnormal histo-pathological group = 36/562, 6.4 %; normal group = 48/16968, 0.3 %; *P* = 0.001), and 58 (abnormal histo-pathological group = 88/562, 15.7 %; normal group = 152/16968, 0.9 %; *P* = 0.001) were detected significantly more frequently in the abnormal histo-pathological group compared with the control group. In addition, HPV-31 (abnormal histo-pathological group = 3/562, 0.5 %; normal group = 36/16968, 0.21 %; *P* = 0.002), 51 (abnormal histo-pathological group = 8/562, 1.4 %; normal group = 52/1402, 0.3 %; *P* = 0.001), 61 (abnormal histo-pathological group = 3/562, 0.5 %; normal group = 24/16968, 0.1 %; *P* = 0.05), 66 (abnormal histo-pathological group = 3/562, 0.5 %; normal group = 24/16968, 0.1 %; *P* = 0.05), and 68 (abnormal histo-pathological group = 10/562, 1.8 %; normal group = 52/16968, 0.3 %; *P* = 0.001) were significantly more common in the normal group compared with the abnormal histo-pathological group (Table 4).

**Table 4:** Prevalence of Human Papillomavirus (HPV) and its genotypes among normal and histo-pathological abnormal cases with Single or Multiple genotypes infection

	Single infection			Multiple infection		
Variable	Normal	Abnormal	*P*-value	Control	Abnormal	*P*-value
	(*n* = 16968)	(*n* = 930)		(*n* = 16968)	(*n* = 930)	
Overall^a^	8.3 (7.9–8.7)	60.4 (58.8–62.0)	0.001	1.8 (1.6–2.0)	25.4 (22.6–28.2)	0.001
Unidentified^a^	0.7 (0.6–0.8)	7.3 (5.6–9.0)	0.001	-	-	
HPV-16^a^	1.6 (1.4–1.8)	14.6 (12.3–16.9)	0.001	0.6 (0.5–0.7)	9.9 (8.0–11.8)	0.001
HPV-52^a^	1.6 (1.4–1.8)	10.3 (8.4–12.2)	0.001	0.6 (0.5–0.7)	7.7 (6.0–9.4)	0.001
HPV-58^a^	0.9 (0.8–1.0)	9.5 (7.6–11.4)	0.001	0.5 (0.4–0.6)	5.6 (4.1–7.1)	0.001
HPV-18	84 (0.5)	48 (5.2)	0.001	52 (0.3)	36 (3.9)	0.001
HPV-68	52 (0.3)	10 (1.1)	0.002	32 (0.2)	28 (3.0)	0.001
HPV-51	52 (0.3)	8 (0.9)	0.012	40 (0.2)	20 (2.1)	0.001
HPV-33	48 (0.3)	36 (3.9)	0.001	32 (0.2)	8 (0.9)	0.01
HPV-39	48 (0.3)	12 (1.3)	0.001	28 (0.2)	28 (3.0)	0.001
HPV-31	36 (0.2)	3 (0.3)	0.45	12 (0.07)	12 (1.3)	0.001
HPV-59	28 (0.2)	12 (1.3)	0.001	12 (0.07)	4 (0.4)	0.008
HPV-56	20 (0.1)	-		20 (0.1)	16 (1.7)	0.001
HPV-45	4 (0.02)	-		-	8 (0.9)	
HPV-35	3 (0.02)	-		6 (0.03)	3 (0.3)	0.008
PHR-HPV						
HPV-53	88 (0.5)	24 (2.6)	0.001	40 (0.2)	24 (2.6)	0.001
HPV-66	24 (0.1)	3 (0.3)	0.16	20 (0.1)	20 (2.1)	0.001
HPV-81	20 (0.1)	11 (1.2)	0.001	32 (0.2)	44 (4.7)	0.001
LR-HPV						
HPV-61	24 (0.1)	3 (0.3)	0.16	-	-	
HPV-11	-	-		24 (0.1)	8 (0.9)	0.001
HPV-6	-	-		12 (0.07)	4 (0.4)	0.008
HPV-42	-	-		5 (0.03)	4 (0.4)	0.001
HPV-55	4 (0.02)	3 (0.3)		-	-	
HPV-40	4 (0.02)	-			-	
HPV-44	4 (0.02)	-		4 (0.02)	4 (0.4)	0.001
HPV-43	3 (0.01)	-		4 (0.02)	8 (0.9)	0.001
Unclassified						
HPV-82	7 (0.04)	-		-	-	
HPV-71	3 (0.02)	-		-	-	
HPV-70	2 (0.01)	-		-	-	
HPV-83	2 (0.01)	-		-	-	
HPV-67	1 (0.005)	-		-	-	
HPV-69	1 (0.005)	-		-	-	

*n* number, ^a^95 % Confidence interval

**Fig. 3:**
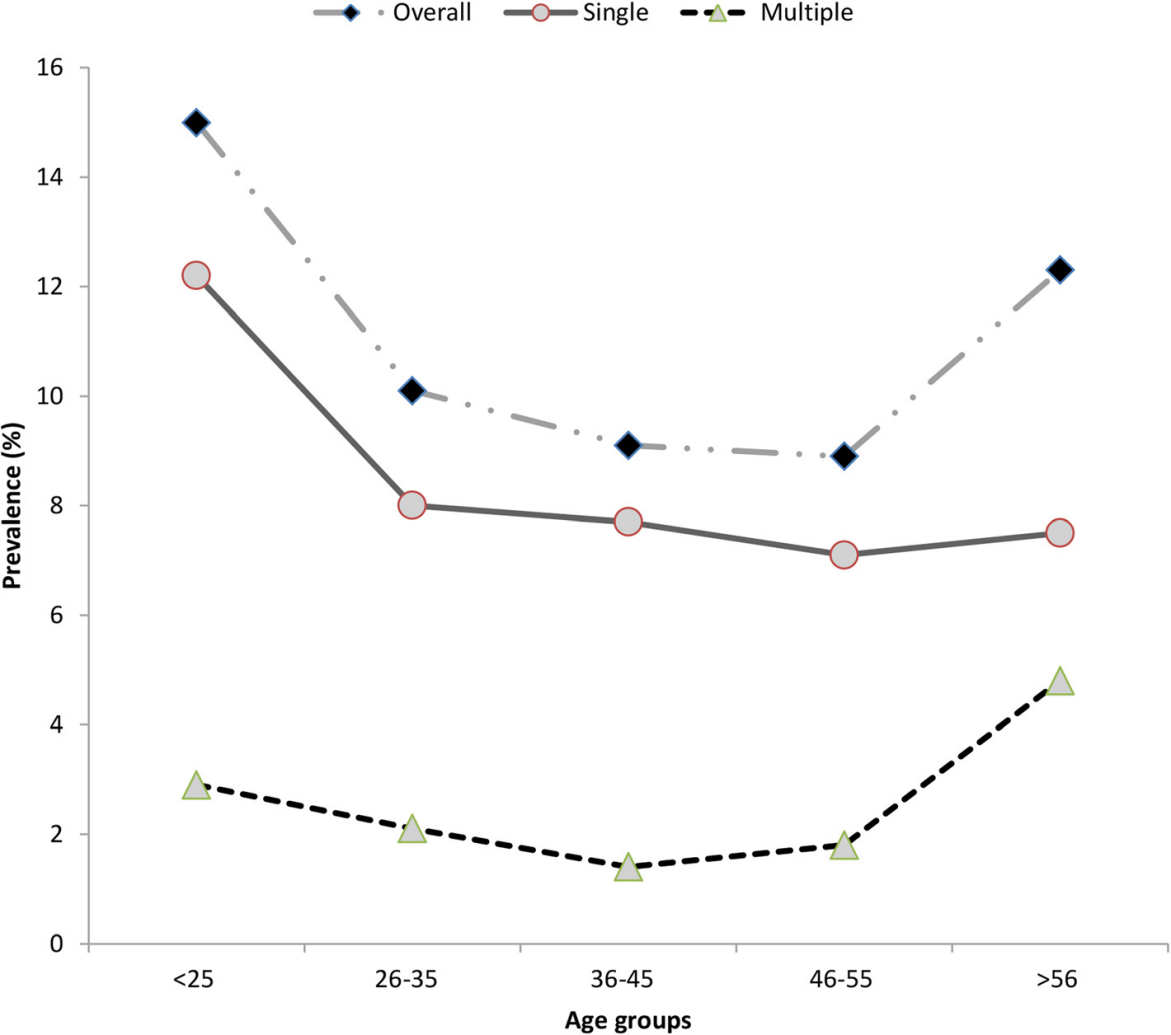
Age-specific HPV prevalence among normal cases

**Fig. 4:**
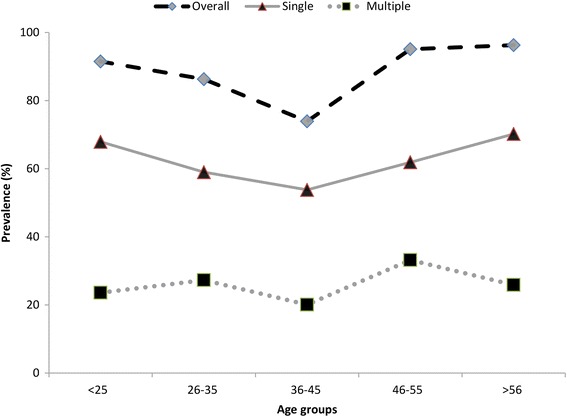
Age-specific HPV prevalence among histo-pathological abnormal cases

To determine the possible variation in HPV genotype distribution among the various age groups, the abnormal histo-pathological and normal groups were stratified into three groups according to age. Correlation analysis revealed that HPV prevalence was more complex in the younger and older participants compared with the middle age group (Figs. 3 and 4). The highest overall, HR-HPV and multiple HPV prevalence was observed in the <35 years age group, and it declined thereafter with increasing age. A less pronounced second peak in prevalence was observed for the normal women in the older age group (>46 years). However, overall, high-risk and multiple-genotype infections among the abnormal histo-pathological women exhibited two peaks in prevalence, with the first peaks of 88.9, 63.5 and 25.5 %, respectively, occurring at <35 years and the second peaks of 95.5, 64.3 and 31.2 % occurring at >46 years. Overall, we did not find significant differences among the various age groups in HPV genotype distribution. Correlation analysis revealed that the HPV genotype prevalence’s were highly correlated in single and multiple infections among the younger women (<35 years) and older women in both the normal and abnormal histo-pathological groups. However, HPV-16 was more frequent in the younger women (<35 years) in both the normal and abnormal histo-pathological groups. The prevalence’s of other genotypes, such as HPV-52, 58, 68, 33, 53, and 66, were found vary among the different age groups and varying histo-pathological grades (Table 5).

**Table 5:** Prevalence of Human Papillomavirus (HPV) genotypes in according to histo-pathological result among various Age groups

	Normal cases				Histo-pathologically abnormal cases			
Variables	>35 years (*N* = 5978)	36–45 years (*n* = 6534)	<46 years (*n* = 4456)	*P*-value	>35 years (*n* = 326)	36–45 years (*n* = 318)	<46 years (*n* = 286)	*P*-Value
Overall	721 (12.1)	592 (9.1)	443 (9.9)	0.001	290 (88.9)	235 (73.9)	273 (95.4)	0.045
Single	579 (9.7)	502 (7.7)	321 (7.2)	0.001	207 (63.5)	171 (53.8)	184 (64.3)	0.9
Multiple	142 (2.4)	90 (1.4)	122 (2.7)	0.44	83 (25.5)	64 (20.1)	89 (31.2)	0.13
HR-HPV	1087							
HPV-16	160 (2.7)	132 (2.0)	88 (2.0)	0.018	100 (30.7)	80 (25.1)	48 (16.8)	0.001
HPV-52	153 (2.5)	119 (1.8)	120 (2.7)	0.003	63 (19.3)	61 (19.2)	16 (5.6)	0.001
HPV-58	98 (1.6)	69 (1.0)	65 (1.4)	0.016	52 (15.5)	80 (25.1)	36 (12.6)	0.37
HPV-18	64 (1.1)	41 (0.6)	31 (0.7)	0.022	22 (6.7)	37 (11.6)	25 (8.7)	0.35
HPV-68	40 (0.7)	31 (0.5)	13 (0.3)	0.024	10 (3.1)	25 (7.9)	3 (1.0)	0.27
HPV-33	44 (0.7)	24 (0.4)	12 (0.3)	0.001	20 (6.1)	24 (7.5)	-	
HPV-51	29 (0.5)	43 (0.6)	20 (0.4)	0.25	12 (3.7)	16 (5.0)	-	
HPV-56	12 (0.2)	16 (0.2)	10 (0.2)	0.87	8 (2.4)	-	8 (2.8)	
HPV-31	10 (0.2)	15 (0.2)	23 (0.5)	0.002	8 (2.4)	6 (1.9)	1 (0.3)	
HPV-59	10 (0.2)	21 (0.3)	9 (0.2)		5 (1.5)	3 (0.9)	8 (2.8)	
HPV-39	8 (0.1)	32 (0.5)	16 (0.3)		8 (2.4)	18 (5.7)	14 (4.9)	
HPV-35	4 (0.07)	2 (0.03)	3 (0.07)		-	3 (0.9)	-	
HPV-45	1 (0.02)	3 (0.04)	-		2 (0.6)	-	6 (2.1)	
PHR-HPV	132							
HPV-53	28 (0.5)	56 (0.8)	44 (1.0)	0.003	16 (4.9)	16 (5.0)	16 (5.6)	0.7
HPV-66	23 (0.4)	11 (0.2)	10 (0.2)	0.05	13 (4.0)	6 (1.9)	4 (1.4)	
HPV-81	20 (0.3)	12 (0.2)	20 (0.4)	0.042	-	22 (6.9)	15 (5.2)	
Others	183				-	-	-	
HPV-83	-	2 (0.03)	-		-	-	-	
HPV-69	-	1 (0.01)	-		-	-	-	
HPV-70	-	2 (0.03)	-		-	-	-	
HPV-71	-	-	3 (0.07)		-	-	-	
HPV-82	4 (0.05)	-	3 (0.07)		-	-	-	
HPV-67	1 (0.02)	-	-		-	-	-	
HPV-61	11 (0.2)	7 (0.1)	6 (0.1)	0.5	3 (0.9)	-	-	
HPV-11	10 (0.2)	7 (0.1)	7 (0.1)	0.63	-	-	8 (2.8)	
HPV-6	5 (0.08)	7 (0.1)	-		-	-	4 (1.4)	
HPV-40	4 (0.07)	-	-					
HPV-44	4 (0.07)	4 (0.06)	-		-	4 (1.2)	-	
HPV-43	2 (0.03)	-	5 (0.1)		-	3 (0.9)	5 (1.7)	
HPV-42	1 (0.02)	3 (0.04)	1 (0.02)		-	4 (1.2)	-	
HPV-55	-	4 (0.06)	-		-	3 (0.9)	-	
Unidentified	44 (0.7)	76 (1.2)	8 (0.2)	0.001	28 (8.6)	32 (10.1)	8 (2.8)	0.008

## Discussion

Many studies have reported the HPV prevalence and its genotype distributions in various provinces of P. R. China [22, 23]. However, no relevant epidemiological data are available for Yunnan province. To the best of our knowledge, the current study is the first to report the epidemiological prevalence and distribution of HPV genotypes in various region of Yunnan. Samples screened in this study were obtained from different ethnic populations from different geographic regions of Yunnan province. Therefore, the results of this study provide precise estimations of the HPV prevalence and genotype distributions in histo-pathological abnormal and normal cervical samples obtained from women throughout Yunnan province.

In this study, the overall HPV infection rate of 14.0 % was higher than the documented rates in neighbouring countries (6.2 % in Southeast Asia, 6.6 % in south central Asia and 8.0 % in other Asian countries [15, 24]. However, reports from some regions of mainland China have stated that the overall prevalence is 18.4 % in Shenzhen city [25], 13.3 % in Zhejiang province [26] and 14.8 % in Shanxi province [27], in agreement with our reported HPV prevalence. HPV prevalence has been documented to vary in different geographic regions, e.g., it is 8.1 % in Europe, 11.3 % in North America, 22.1 % in Africa and 8.0 % in Asia [15]. Here, we found that its prevalence was significantly higher among the south-western women (19.9 %) than the women from other four regions. The HPV prevalence in the south-western and north-western region was in line with the previously reported infection rate for north-western region of Yunnan (18.4 %) [27, 28]. However, the HR-HPV prevalence in the south-western women was very high compared with Zhejiang (10.2 %) and Shanxi (12.2 %) [29, 30]. The LR-HPV prevalence rates have been reported to be 3.8 and 4.4 % in Shanxi and Taiwan, respectively [29], which is in accordance with our identified LR-HPV prevalence of 4.1 % among the north-western region women. Comparatively, the calculated LR-HPV prevalence rate among the central, south-western, south-eastern and north-eastern regions women is similar to those in areas with lower LR-HPV prevalence rates in China, such as Shandong and Tibet [29, 31].

The distribution of HPV genotypes varies significantly in different geographic locations worldwide [15]. HPV-16 is the most frequently detected genotype in all regions of the world, followed by HPV-58 and 52 in Asia [24], HPV-58 in South America and HPV-31 in Europe [5]. In China, HPV-16 is the most prevalent genotype, followed by HPV-52 and 58 [26]. Moreover, HPV-33 and 31 are highly prevalent in some regions [27]. Such variation was also observed in this study, with HPV-52 being the most prevalent genotype in the central and north-east regions, followed by HPV-16 and 58. Interestingly HPV-18 is the 3rd most prevalent genotype in the north-east region only. In the north-west and south-east regions, HPV-16 is the most frequent, followed by HPV-52 and 58. Further, HPV-16 is most common in the south-west region, followed by HPV-58 and 52 (Table 5).

Our study findings do not support previously reported data that infection with multiple genotypes increases the risk of cervical cancer. Infection with multiple genotypes was significantly more frequent in the CINI and CIN2 samples. However, the prevalence this type of infection abruptly decreased with progression to CIN3 or cervical cancer. Further, infections with a single HR-HPV genotype resulted in an increase in cervical lesion progression. This finding might indicate that there is a lower risk of developing malignancy with multiple genotype infections compared with single genotype infections [32, 33] (Table 1).

Analysis of the specific HPV genotype distribution in the abnormal histo-pathological group indicated that it was highly variable in the histo-pathological abnormal lesions. HPV-16 (45.4 %) was the most frequently detected genotype in SCC, followed by HPV-58 (18.2 %), 18 (18.2 %), and 52 (15.9 %). Interestingly, 8 SCC patients were also found to have a multiple genotype infection (co-infection of HPV-59 and 66 with HPV-52 and 58, and 18). These findings are in complete agreement with previously reported data, indicating that HPV-16, 18, 52, and 58 are most frequently present in cervical cancer [9, 34]. HPV-16, 58, 18 and 52 were also frequent in the CIN3 samples, which is consistent with previous reports [25, 35]. All of these genotypes are considered to be highly oncogenic and are the most frequently detected HPV genotypes worldwide. Previous studies conducted in China and some other parts of the world have indicated that HPV-52 is the most frequent genotype in Asia, and particularly in China [25, 30]. In this study, HPV-52 was also a predominant genotype; however, its prevalence was higher in CIN1 and CIN2 samples compared with that in advanced cervical lesions. Based on these observations, infection with HPV-16, 58, and 18 might be associated with an elevated risk of cervical cancer development in the infected population. However, the declining trend in HPV-52 prevalence in advanced cervical lesions might be due to host immune factors or geographic or environmental factors [36]. In this study, we found high prevalence of other HR-HPV genotypes, such as HPV-33, 31, 39, 51, and 68, in the abnormal histo-pathological group. These genotypes were more common in the CIN1, CIN2 and CIN3 lesions than in SCC. HPV-31 and 33 are highly carcinogenic genotypes that are present worldwide [33, 37]. However, in this study, we did not find them to be highly carcinogenic, which may be due to variable geographic factors, biological factors, lifestyle, or human and viral genetic factors. Further, we found a high prevalence of PHR-HPV, particularly HPV-53 and 81 and the LR-HPV genotypes HPV-6 and 11. However, these genotypes were only identified in multiple genotype infections. These findings are in line with previously reported data [38]. In this study, the prevalence of HR-HPV was increased in samples with advanced histo-pathological grades. The incidence of HR-HPV infection, and particularly that of single genotype infection, was very high in the abnormal histo-pathological group compared with that in the normal group. These findings are in accordance with those of Castle et al. [39]. On the basis of these observations, we suggest that attention should be paid to the histo-pathological outcomes of CIN2, CIN3 and higher-grade lesions, even in the absence of specific genotype information.

In this study, we also evaluated the HPV genotype distributions among different age groups. HPV-16 was more frequent in women less than 35 years old in both the normal and abnormal cytology groups. However, the distributions of the other genotypes varied among the different age groups with lesions of differing cytology grades. HPV-52 and 18 showed double-peak prevalence in the normal cytology group. The prevalence of HPV-52 in the abnormal cytology group decreased with increasing age; however, that of HPV-18 increased with increasing age in this group. Such variation has also been observed for other genotypes, as shown in Table 4. These findings indicate that various age groups possess different genotypes in lesions with differing cytological grades [40]. Thus, it is very important to determine the potential roles of the different HPV genotypes in carcinogenesis development among various age groups and to further determine their potential risk and contribution to carcinogenesis in future studies.

The results of this study suggest that cervical screening is vital for women, particularly minority ethnic women, to prevent HPV-related cervical cancer [41]. An important link between HPV infection and development of cervical neoplasia has been identified in this study. Thus, the timely detection of HPV infection would be very helpful for preventing disease progression, considering that its early detection in patients with cervical lesions has been well established to result in a decrease in the rate of cancer development [42].

There are some limitations of this study that must be considered when interpreting our findings. Yunnan includes 26 state-certified ethnic minorities who live in different regions of the province. Most of them do not want to participate in epidemiological studies due to their ethnic beliefs. In this study, we focused only on those women who visited a public hospital with routine gynaecological examination.

## Conclusion

This study provides epidemiological estimates of HPV genotype distributions in different regions of the province and further reports the prevalence of these genotypes in neoplastic lesions with differing cytological grades. We have found that overall, HPV-16, 52, 58, and 18 are the most common HR-HPV genotypes. However, the prevalence rates of these genotypes significantly differ in different regions of the province. PHR-HPV-53 is also a predominant genotype in some regions. These variations could be due to the large number of ethnic populations residing in the province. A vaccine currently under trial has been formulated for HPV-16, 18, 11, and 6, and it will be introduced nationally in the coming years. However, HPV-52 and 58 are the predominant genotypes in several regions. Thus, based on our observations, a new tetravalent vaccine may be more effective than a bivalent vaccine in China, and particularly in Yunnan province. Further, due to large-scale geographic variation in HPV genotype distribution, a future large-scale, multi-ethnic population-based study must be conducted to obtain comprehensive information on the prevalence and genotype distribution of HPV in various Chinese ethnic populations.

## Abbreviations

HPV, human papillomavirus; HR-HPV, high risk-HPV; PHR-HPV, potential high risk-HPV; LR-HPV, low risk-HPV; CIN, cervical intraepithelial neoplasia; CC, cervical cancer.
